# Gut microbiota alterations and their correlation with constipation in older adults with Alzheimer’s disease or mild cognitive impairment: A cross-sectional study

**DOI:** 10.1097/MD.0000000000050227

**Published:** 2026-08-14

**Authors:** Kai-Yong Huang, Yi-Yu Wei, Shu-Ting Zhang, Liu-Chun He, Jing Zhou, Lan-Shu Zhou, Hua-Bin Su, Guo-Dong Lu

**Affiliations:** a Department of Occupational Health and Occupational Medicine, School of Public Health, Guangxi Medical University, Nanning, Guangxi, China; b Guangxi Colleges and Universities Key Laboratory of Prevention and Control of Highly Prevalent Diseases, School of Public Health, Guangxi Medical University, Nanning, Guangxi, China; c Guangxi Key Laboratory of Environment and Health Research, School of Public Health, Guangxi Medical University, Nanning, Guangxi, China; d Department of Physiology, School of Basic Medical Sciences, Guangxi Medical University, Nanning, Guangxi, China; e Ministry of Science and Education, Jiangbin Hospital of Guangxi Zhuang Autonomous Region, Nanning, Guangxi, China; f Department of Occupational Health and Toxicology, School of the Public Health, Fudan University, Shanghai, China.

**Keywords:** Alzheimer’s disease, constipation, gut microbiota, mild cognitive impairment, older adult

## Abstract

Previous studies have suggested a link between gut microbiota, constipation and Alzheimer’s disease (AD) as well as mild cognitive impairment (MCI). In this study, we aimed to investigate the changes in the gut microbiota and their relationship with constipation in patients with AD and MCI. A total of 90 participants (30 with AD, 30 with MCI, and 30 normal controls) were recruited from the community-based Nanning Community Elderly Population cohort. We collected fresh fecal samples for analysis and compared the differences in the gut microbiota among the groups. We also categorized these 90 participants into a constipation group and a non-constipation (normal) group using the Rome IV diagnostic criteria, allowing for a comparison of gut microbiota between these 2 groups. There were significant differences in alpha and beta diversity of the gut microbiota among the AD, MCI, and control groups, as well as between the constipation and non-constipation groups. At the genus level, the relative abundance of *Lachnospira* was decreased in both the AD group and the constipation group, while the relative abundance of *Paeniclostridium* was increased in both the MCI group and the constipation group. Our findings suggest that changes in the gut microbiota in patients with AD or MCI are partially consistent with those observed in older adults with constipation and may be involved in the pathogenesis of AD.

## 1. Introduction

In 2019, the number of people living with dementia worldwide was estimated at 57 million, and it is projected to increase to 153 million by 2050.^[1]^ The proportion of people with dementia has increased over time in lower-income countries due to a greater percentage increase in longevity than in high-income countries.^[2]^ Alzheimer’s disease (AD) is the most common form of dementia among older individuals, manifesting not only in cognitive impairment as well as psychiatric symptoms such as depression and anxiety.^[3]^ Despite decades of research, the comprehensive pathogenesis of AD remains to be elucidated and so far, no effective treatment has been developed.^[4]^ Furthermore, due to the high incidence of this disease alongside the lack of effective drugs and the poor prognosis, AD imposes a huge economic and mental burden on families and society.^[5]^ The existing evidence indicates that many factors, such as aging, inflammation, hypertension, physical inactivity, diabetes, social isolation, obesity, and insulin resistance, are risk factors for AD.^[1]^ Mild cognitive impairment (MCI), a diagnostic entity defined as an intermediate stage between subjective cognitive decline and dementia, does not significantly affect daily life.^[6,7]^ Around 30%–50% of older adults with MCI progress to dementia within 5 years, compared with 3% of those without MCI at the same age.^[8,9]^ However, some individuals with MCI can remain stable or return to normal over time. Early detection of MCI is an essential step to slow the progression to AD in a timely manner.

More recently, the influence of the gut microbiota on central nervous system (CNS) function: often referred to as the gut–brain axis: has received significant attention.^[4]^ The gut microbiota is considered as an “organ,” producing numerous metabolites that interact with the host’s physiology and affect functions in the local intestine and distant brain.^[10]^ Numerous studies have demonstrated that alterations in the gut microbiota have been associated with neurological conditions including autism spectrum disorder, multiple sclerosis, Parkinson’s disease, AD, and MCI.^[3–5]^ A previous study showed that the diversity of the gut microbiota is reduced in fecal samples from participants with AD.^[4]^ At the phylum level, the authors noted decreased abundance of *Firmicutes* and *Actinobacteria* but increased abundance of *Bacteroidetes* in patients with AD. More importantly, patients with MCI have similar alterations in the patients with AD in the gut microbiota,^[11]^ which may provide a new idea for the early-stage diagnosis of AD. Similarly, differences in the gut microbiota between AD and wild-type mice of the same age have also been observed.^[5]^

Constipation is a common gastrointestinal condition in older people: The global pooled prevalence is 10%–15% in community-dwelling older adults.^[12]^ In recent years, researchers have revealed that constipation is associated with various neurological and neurodegenerative diseases, including Parkinson’s disease, Lewy-body dementia, and AD.^[12–15]^ In our previous studies, we have found that constipation is associated with increased risk of MCI in community-living older adults.^[16,17]^ Accumulating evidence highlights the link between constipation and alterations of the gut microbiota as a risk factor of neurodegenerative diseases.^[18]^ The gut–brain axis is believed to play a crucial role in the relationship between CNS disorders and the onset and progression of constipation.^[12]^ Constipation can result in gut dysbiosis, which further exacerbates imbalances in the gut–brain axis and potentially contributes to other neurological conditions.^[19]^ This axis may provide a novel research angle to understand the development of neurological diseases.

Although researchers have examined alterations of the gut microbiota in patients with AD and MCI, the results have been inconsistent. A relatively unexplored area is alterations of the gut microbiota in patients with constipation. There is no report on whether alterations of the gut microbiota caused by constipation are identical to or associated with alterations of the gut microbiota observed in patients with AD and MCI. Therefore, we aimed to investigate alterations of the gut microbiota and their correlation with constipation in older adults with AD or MCI.

## 2. Materials and methods

### 2.1. Participants

A total of 90 participants (30 with AD, 30 with MCI, and 30 normal controls) were recruited from the Nanning Community Elderly Population cohort, a community-based cohort in Nanning, China. These 90 elderly individuals were also divided into a constipation group and a non-constipation (normal) group based on the Rome IV diagnostic criteria. This cross-sectional study was conducted at Guangxi Medical University, Nanning, China, from July 2021 to November 2021. This study adhered to the principles of the World Medical Association’s Declaration of Helsinki and was approved by the Medical Ethics Committee of Guangxi Medical University (protocol number 20210132). All participants were informed about the research procedure and signed a written consent form before they were included in the study.

The inclusion criteria for this study were community residents who were: aged 60 years or older; a local resident for at least 1 year; for the older adults with MCI or AD, diagnosed based on the 2011 National Institute of Aging and Alzheimer’s Association criteria,^[20]^ and for the normal controls, no evidence of cognitive deficits based on neuropsychological tests; and voluntary participation and able to cooperate for the physical examination, neuropsychological tests, and fecal sample collection. The exclusion criteria were those who: had been diagnosed with dementia or other major mental diseases (including Parkinson’s disease, epilepsy, and any acute phase of brain infectious diseases); had taken antibiotics, probiotics, laxatives, steroid hormones, corticosteroids, immunostimulants, immunosuppressants, or medications that affect gastrointestinal motility within the past 3 months; had severe gastrointestinal diseases, such as diarrhea, irritable bowel syndrome, inflammatory bowel disease, intestinal malignancy, and Crohn’s disease, which can influence the gut microbiota; had a history of major gastrointestinal tract surgery in past 5 years; and could not cooperate in collecting feces.

### 2.2. Demographics and assessment of covariates

Well-trained research staff members used a questionnaire to collect information, including socio-demographic characteristics (age, gender, race, profession, body mass index, marital status, education, annual household income, and co-living situation), medical history (hypertension, diabetes, apoplexy, digestive system diseases, mental diseases, and tumors), lifestyle and behavior (drinking, smoking, physical exercise, and social activities), and medications (digestive system drugs, antibiotics, and hormone therapy drugs).

### 2.3. Neuropsychological assessment

Cognitive function was assessed using the Chinese version of the Mini-Mental State Examination (MMSE).^[21]^ After completing the MMSE, participants were assessed with the Activity of Daily Living Scale to evaluate social functioning,^[22]^ and the Clinical Dementia Rating Scale to examine the severity of dementia and cognitive decline.^[23]^ The Clinical Dementia Rating Scale covers 6 cognitive, behavioral, and functional aspects, namely memory, orientation, judgment and problem-solving, community affairs, home and hobby performance, and personal care.^[24]^ An expert panel consisting of 2 neurologists and neuropsychologists administered the comprehensive neuropsychological tests to the participants. All participants underwent the same neuropsychological testing.

### 2.4. Constipation diagnosis

Constipation was assessed by the expert panel using a questionnaire according to the Rome IV criteria.^[25]^ The criteria had to be fulfilled for the last 3 months and symptom onset had to be at least 6 months prior to diagnosis: Must include 2 or more of the following: straining during more than one fourth (25%) of defecation; lumpy or hard stools (Bristol Stool Form Scale 1–2) during more than one fourth (25%) of defecation; sensation of incomplete evacuation during more than one fourth (25%) of defecation; sensation of anorectal obstruction/blockage during more than one fourth (25%) of defecation; manual maneuvers to facilitate more than one fourth (25%) of defecation (e.g., digital evacuation, support of the pelvic floor); fewer than 3 spontaneous bowel movements per week. Loose stools are rarely present without the use of laxatives. Insufficient criteria for irritable bowel syndrome with constipation.^[25]^

### 2.5. Fecal sample collection and DNA extraction

The aseptic fecal sample collection containers were delivered with detailed user guidance to the participants before the visit. All participants involved in the study resided at home, where fecal samples were collected. The participants were required to collect their fasting fecal samples in the morning and send their samples to the local community hospital within 1 hour. The returned samples were weighed, subsampled (~100 mg) into prepared sterile bead-beating tubes, and stored at −80°C until analysis.^[4]^ The fecal bacterial DNA was extracted with the cetyltrimethylammonium bromide method. The DNA was assessed with 1% agarose gel electrophoresis, and the DNA concentration and purity were evaluated with a NanoDrop One spectrophotometer (Thermo Fisher Scientific, Waltham, MA, USA). The 260 nm to 280 nm absorbance ratio was used to assess protein contamination, and the 260 nm to 230 nm absorbance ratio was used to assess guanidine contamination. Each extract was averaged across numerous measurements to minimize mistakes.^[24,26]^

### 2.6. Polymerase Chain Reaction (PCR) amplification and illumina MiSeq sequencing

Illumina-indexed amplicons were generated using PCR amplification of the V4 region of bacterial 16S ribosomal RNA (rRNA) gene using the 515R (5′-GTGCCAGCMGCCGCGGTAA-3′) and 806R (5′-GGACTACHVGGGTWTCTAAT-3′) primers. PCR was performed twice with Phusion® High-Fidelity PCR Master Mix (New England Biolabs, Ipswich, MA, USA). The PCR products were evaluated with 2% agarose gel electrophoresis.^[27]^ The PCR products were mixed in equidensity ratios. Then, the mixture of PCR products was purified with the Qiagen Gel Extraction Kit (Qiagen, Hilden, Germany). All samples that passed the quality control analysis were utilized for library preparation. Sequencing libraries were generated using the NEBNext Ultra DNA Library Prep kit for Illumina (New England Biolabs) following the manufacturer’s recommendations, and index codes were added. The library quality was assessed on the Qubit@ 2.0 Fluorometer (Thermo Fisher Scientific) and Agilent Bioanalyzer 2100 system (Agilent Technologies). Finally, the library was sequenced on the Illumina NovaSeq6000 sequencing platform of Beijing Novogene Co. Ltd. (Beijing, China).^[28]^

### 2.7. Processing of sequencing data

The raw 16S rRNA gene sequencing reads were demultiplexed, quality filtered with FASTP (version 0.20.0, Shenzhen Institutes of Advanced Technology, Chinese Academy of Sciences, Shenzhen, China), and merged in FLASH (version 1.2.7, Johns Hopkins University School of Medicine).^[24]^ Sequences with ambiguous base pairings, sequences longer than 275 base pairs (bp), and homopolymers >8 bp were removed. Then, sequences were aligned to the SILVA 16S rRNA gene reference alignment database, and chimeric sequences were identified and removed.^[4]^ Finally, all of the effective tags were clustered into operational taxonomic units (OTUs) using the Uparse software (Uparse v7.0.1001, http://www.drive5.com/uparse/) at 97% sequence similarity and were annotated against the SILVA132 (http://www.arb-silva.de/).^[29]^ Multiple sequence alignment was conducted using the MUSCLE software (version 3.8.31). The OTU abundance information was normalized using a standard sequence number corresponding to the sample with the fewest sequences. All normalized sequences were generated for the downstream analysis.^[29]^

### 2.8. Alpha and beta diversity and linear discriminant analysis effect size (LEfSe)

Microbial diversity was assessed using both alpha and beta diversity metrics. The OTUs were used to assess alpha diversity based on the Chao 1, phylogenetic diversity (PD) whole tree, observed species, and Shannon indices, which were generated with QIIME (version 1.7.0) and displayed with R (version 2.15.3).^[30]^ The Mann–Whitney *U* test was used for statistical comparisons between the groups. Beta diversity, including principal coordinates analysis and non-metric multi-dimensional scaling, was evaluated using Bray–Curtis dissimilarity matrices, computed using the R packages vegan and ade4. Inter-group differences in community composition were assessed using the Adonis function in the vegan package.^[31]^ LEfSe was performed to identify the bacterial taxa that were differentially represented between groups at the genus or higher level. The species-level abundance data were normalized and processed using the Mothur software. Taxa with linear discriminant analysis scores >4 and *P*-values < .05 were considered to be significantly different between the groups.^[31]^

### 2.9. Statistical methods

Normally distributed continuous variables are presented as the mean and standard deviation, while categorical variables are presented as the number (n) and frequency (%). The data were analyzed using SPSS Statistics version 22.0 (IBM Corp., Armonk) and R (version 4.0.2). One-way analysis of variance was used to evaluate between-group differences for the continuous variables. Pearson’s chi-square test were used to determine significant differences for the categorical variables. A two-sided *P*-value of < .05 was considered to indicate a statistically significant difference.

## 3. Results

### 3.1. Clinical and demographic data of older adults with AD or MCI and cognitively normal controls

Table 1 showed the clinical and demographic characteristics of the participants. There were no significant differences between the AD and MCI groups in terms of gender, age, body mass index, years of schooling, ethnicity, current work status, marital status, smoking, drinking, diabetes, and hypertension (all *P *> .05). The AD group had the highest proportion of constipation (66.7%), followed by the MCI and control groups (43.3% and 13.3%, respectively, *P *< .001).

**Table 1:** Clinical and demographic data of older adults with AD and MCI and controls.

Characteristics	AD group (N = 30)	MCI group (N = 30)	Control group (N = 30)	*F*/*χ*^2^	*P*
Gender				1.843	.398
Male	8 (26.7)	11 (36.7)	13 (43.3)		
Female	22 (73.3)	19 (63.3)	17 (56.7)		
Age (Mean ± SD)	78.9 ± 7.3	75.4 ± 8.0	73.8 ± 7.2	3.613	.031
BMI (kg/m^2^)	24.6 ± 2.1	23.9 ± 2.4	24.2 ± 1.9	2.865	.107
Yr of schooling (Mean ± SD)	4.7 ± 4.3	6.5 ± 4.4	6.5 ± 4.0	1.858	.162
Ethnic				3.029	.220
Han	22 (73.3)	24 (80.0)	18 (60.0)		
Zhuang	8 (26.7)	6 (20.0)	12 (40.0)		
Marital status				1.900	.387
Married/cohabitation	15 (50.0)	16 (53.3)	20 (66.7)		
Divorced/widowed/single	15 (50.0)	14 (46.7)	10 (33.3)		
Work status				1.023	.600
Still working	0 (0.0)	1 (3.3)	1 (3.3)		
Not working	30 (100.0)	29 (96.7)	29 (96.7)		
Smoking				1.184	.553
Yes	3 (10.0)	5 (16.7)	6 (20.0)		
No	27 (90.0)	25 (83.3)	24 (80.0)		
Drinking				3.214	.200
Yes	2 (6.7)	5 (16.7)	7 (23.3)		
No	28 (93.3)	25 (83.3)	23 (76.7)		
Diabetes				1.440	.487
Yes	7 (23.3)	4 (13.3)	4 (13.3)		
No	23 (76.7)	26 (86.7)	26 (86.7)		
Hypertension				4.632	.099
Yes	15 (50.0)	12 (40.0)	7 (23.3)		
No	15 (50.0)	18 (60.0)	23 (76.7)		
Constipation				17.715	<.001
Yes	20 (66.7)	13 (43.3)	4 (13.3)		
No	10 (33.3)	17 (56.7)	26 (86.7)		

AD = Alzheimer’s disease, BMI = body mass index, MCI = mild cognitive impairment, SD = standard deviation.

### 3.2. OTUs in the AD, MCI, and control groups

**Figure 1.**
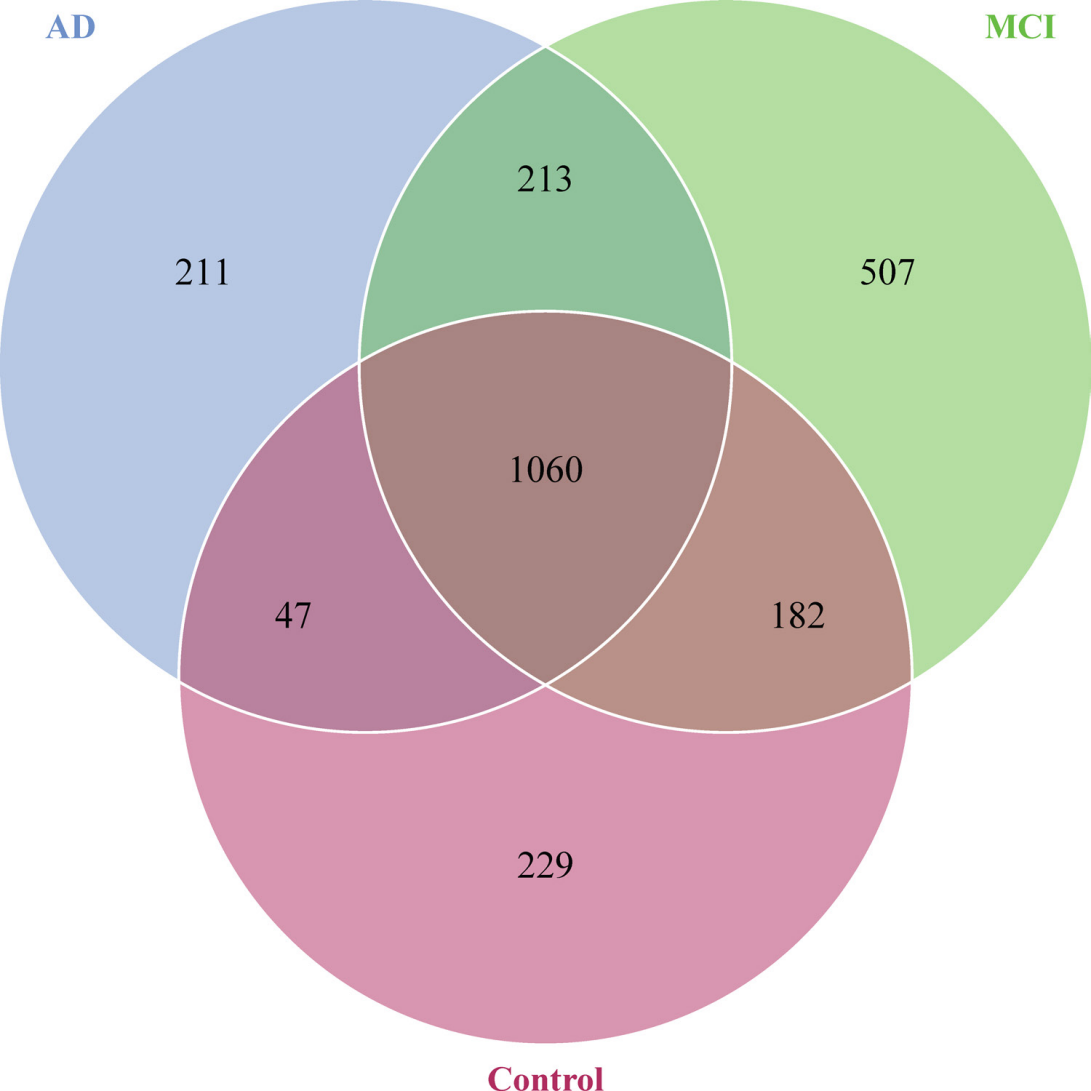
OTUs in AD, MCI and control groups. AD = Alzheimer’s disease, MCI = mild cognitive impairment.

Sequencing of the V3–V4 region of the 16S rRNA gene in the fecal samples generated a total of 7.80 million sequence reads (with a mean ± standard deviation of 86,709 ± 10,615 reads per participant). We identified a total of 2449 OTUs, including 1531 in the AD group, 1962 in the MCI group, and 1518 in the control group (Fig. 1). The OTU dataset was classified into 27 phyla, 45 classes, 97 orders, 176 families, and 401 genera.

### 3.3. Comparison of alpha diversity of the gut microbiota between the AD, MCI, and control groups

**Figure 2.**
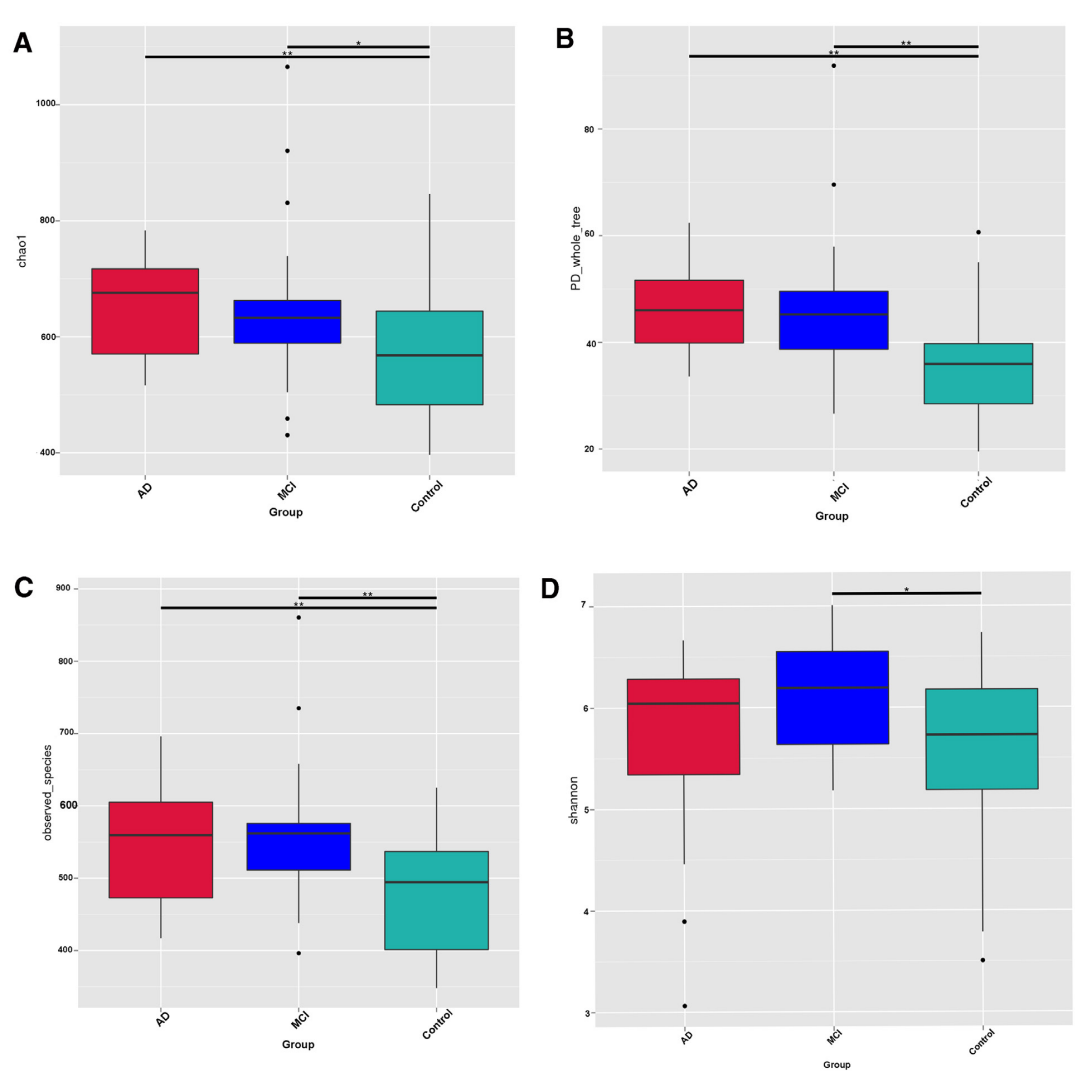
The alpha diversity indices of the fecal microbiota in patients with AD and MCI and controls. (A–D) Box plots depict differences in the fecal alpha diversity indices according to the Chao 1 index, PD whole tree index, observed species index, and Shannon index based on the OUT counts. Each box plot represents the median, interquartile range, minimum, and maximum values. **P < .01; *P < .05. AD = Alzheimer’s disease, MCI = mild cognitive impairment.

The mean community diversity (based on the Chao1 index), PD whole tree index, and observed species index were significantly higher in the AD group compared with the control group (*P *< .05). The mean community diversity of PD whole tree, observed species, and Shannon indices were significantly higher in the MCI group compared with the control group (*P *< .05). However, there was no significant difference in the 4 indices between the AD and MCI groups (*P *> .05, Figure 2A–D).

### 3.4. Comparison of beta diversity of the gut microbiota between the AD, MCI, and control groups

**Figure 3.**
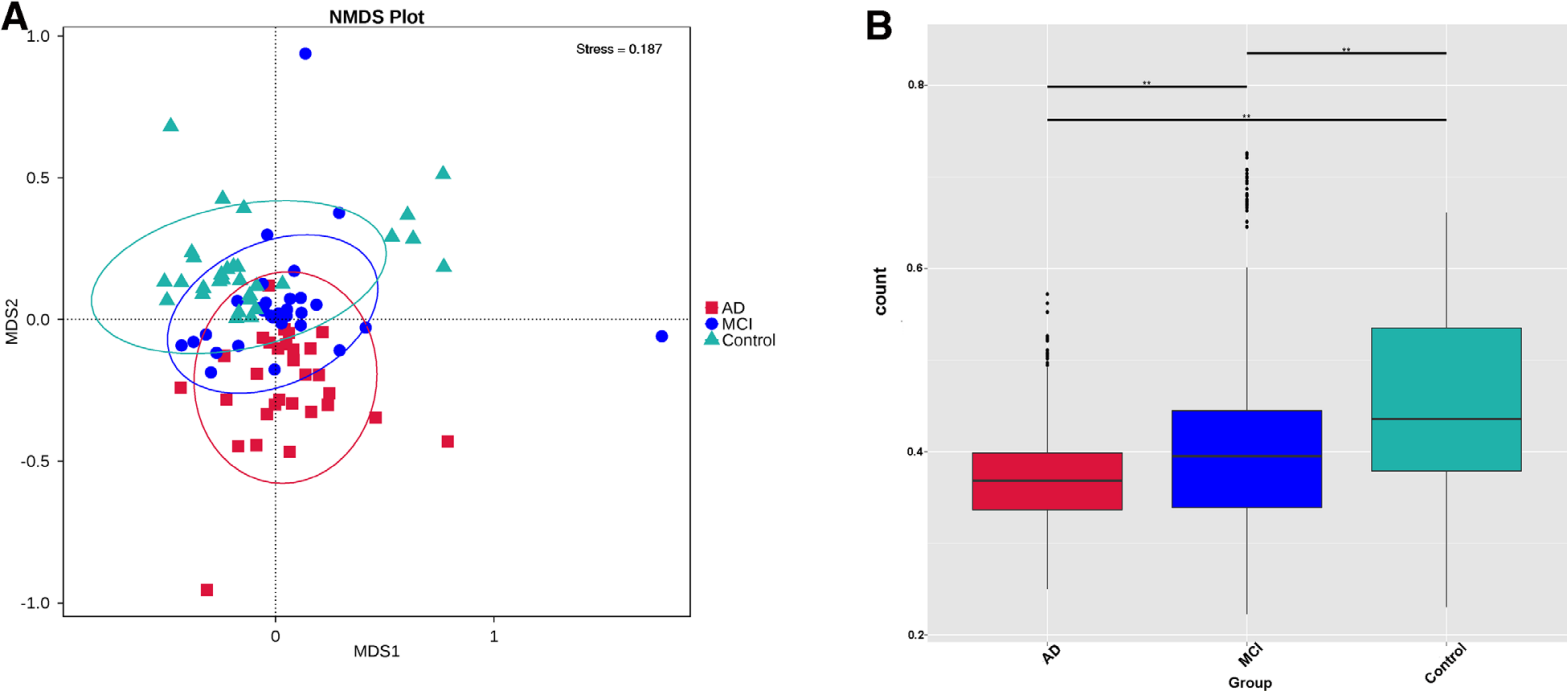
The beta diversity indices of the fecal microbiota in patients with AD and MCI and controls. (A) Non-metric multi-dimensional scaling (NMDS) plot of weighted UniFrac analysis of relative sample OTU composition. NMDS analysis was limited to 2 dimensions, with a stress measurement of 0.187. The stress measurement <0.2 indicated that NMDS could accurately reflect the degree of differences between samples. Each dot represented a scaled measure of the composition of a given participant, and samples from the same group were represented using the same color and shape. (B) Weighted ANOSIM based on the distance matrix of UniFrac dissimilarity of the fecal microbiota communities in the AD, MCI, and control groups. Each box plot represents the median, interquartile range, minimum, and maximum values. **P < .01. AD = Alzheimer’s disease, MCI = mild cognitive impairment.

non-metric multi-dimensional scaling showed a significant difference between the AD, MCI, and control groups in beta diversity (Fig. 3A). Further, we used Anosim to analyze the differences in gut microbial community structure between the 3 groups (Fig. 3B). There was a significant difference between the AD and control groups (ANOSIM *R* = 0.151, *P* < .01), the AD and MCI groups (ANOSIM *R* = 0.084, *P* < .01), and the MCI and control groups (ANOSIM *R* = 0.112, *P* < .01, Figure 3B).

### 3.5. Comparison of LEfSe of the gut microbiota between the AD, MCI, and control groups

**Figure 4.**
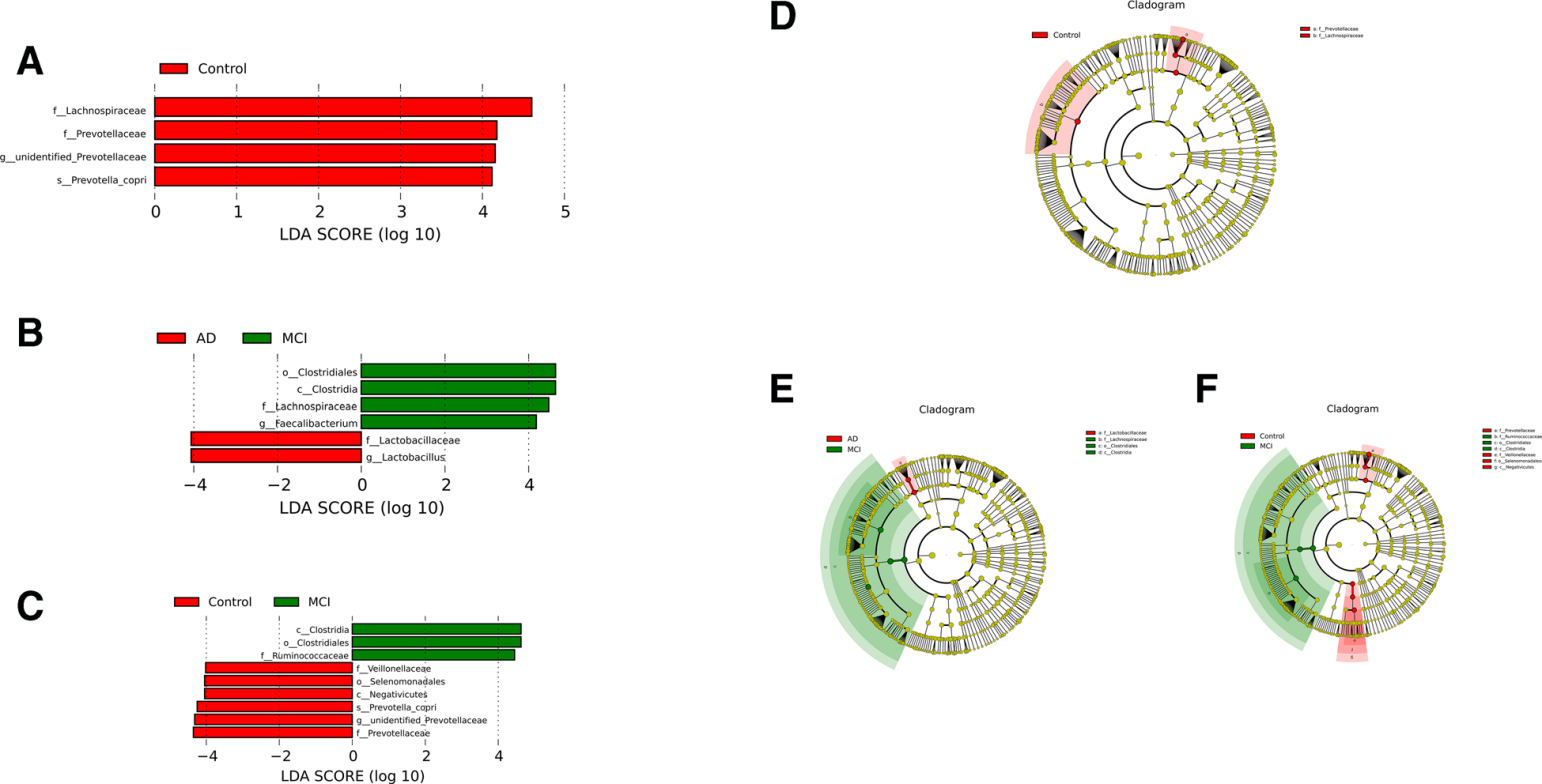
Differences of bacterial taxa among AD, MCI and control groups. (A) The differences in the LDA scores and histogram between AD and control groups. (B) The differences of the LDA scores and histogram for bacterial genera between AD and MCI groups. (C) The differences in LDA scores histogram for bacterial genera between MCI and control groups. (D–F) The cladogram illustrates the phylogenetic distribution of microbial lineages among AD, MCI and control groups. Differently abundant microbiota are listed and marked by different color. The diameter of each circle’s diameter is proportional to the taxon’s abundance. AD = Alzheimer’s disease, MCI = mild cognitive impairment, LDA = linear discriminant analysis.

LEfSe revealed that, compared with the AD group, *f_Lachnospiraceae*, *f_Prevotellaceae*, *g_unidentified_Prevotellaceae*, and *s_Prevotella_copri* were significantly enriched in the control group (Fig. 4A and D). Compared with the MCI group, the AD group showed increased abundance of *f_Lactobacillaceae* and *g_Lactobacillus*, while *o_Clostridiales*, *c_Clostridia*, *f_Lachnospiraceae*, and *g_Faecalibacterium* were enriched in the MCI group (Fig. 4B and E). Compared with the control group, the MCI group presented greater abundance of *c_Clostridia*, *o_Clostridiales*, and *f_Ruminococcaceae*, while the control group showed enrichment of *f_Veillonellaceae*, *o_Selenomonadales*, *c_Negativicutes*, *s_Prevotella_copri*, *f_Prevotellaceae*, and *g_unidentified_Prevotellaceae* (Fig. 4C and F).

### 3.6. Comparison of the gut microbiota genera between the AD, MCI, and control groups

**Figure 5.**
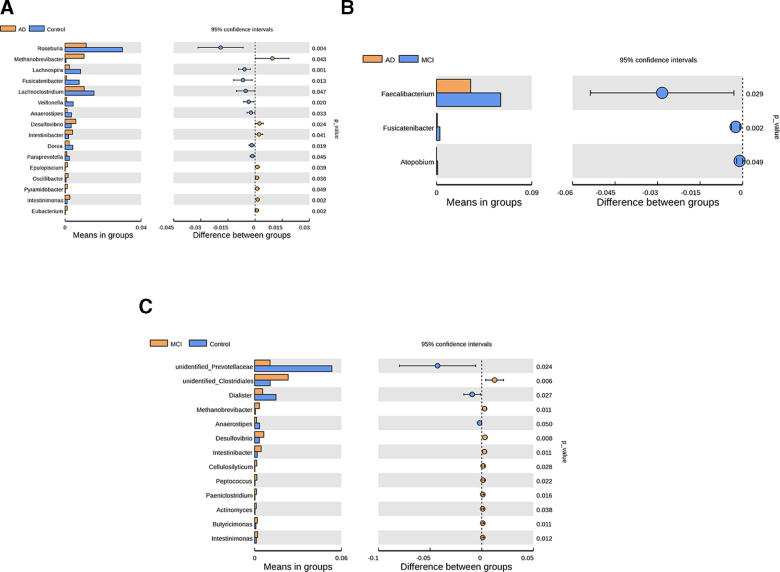
Different fecal microbiota in patients with AD and MCI and controls in genera level. The left histograms represented the mean relative abundance of microbiota in groups, and the right side represented *P* values and the 95% confidence intervals of differences between groups. Significant statistical difference by *t* test (*P* < .05). (A) The differences in abundance at the genus level between AD and control groups. (B) The differences in abundance at the genus level between AD and MCI groups. (C) The differences in abundance at the genus level between MCI and control groups. AD = Alzheimer’s disease, MCI = mild cognitive impairment.

We analyzed the most abundant genera for the AD, MCI, and control groups. Compared with the control group, in the AD group, *Methanobrevibacter*, *Desulfovibrio*, *Intestinibacter*, *Epulopiscium*, and *Oscillibacter* were significantly more abundant, while *Roseburia*, *Lachnospira*, *Fusicatenibacter*, *Lachnoclostridium*, *Veillonella*, *Anaerostipes*, *Dorea*, and *Paraprevotella* were significantly less abundant (all *P* < .05, Figure 5A). Compared with the MCI group, *Faecalibacterium*, *Fusicatenibacter*, and *Atopobium* were significantly less abundant in the AD group (all *P* < .05, Figure 5B). Compared with the control group, *unidentified Clostridiales*, *Methanobrevibacter*, *Desulfovibrio*, *Intestinibacter*, *Cellulosilyticum*, *Peptococcus*, *Paeniclostridium*, *Actinomyces*, *Butyricimonas*, and *Intestinimonas* were significantly more abundant in the MCI group, while *unidentified Prevotellaceae*, *Dialister*, and *Anaerostipes* were significantly less abundant (all *P* < .05, Figure 5C).

### 3.7. Comparison of alpha diversity of the gut microbiota between the constipation and normal groups

**Figure 6.**
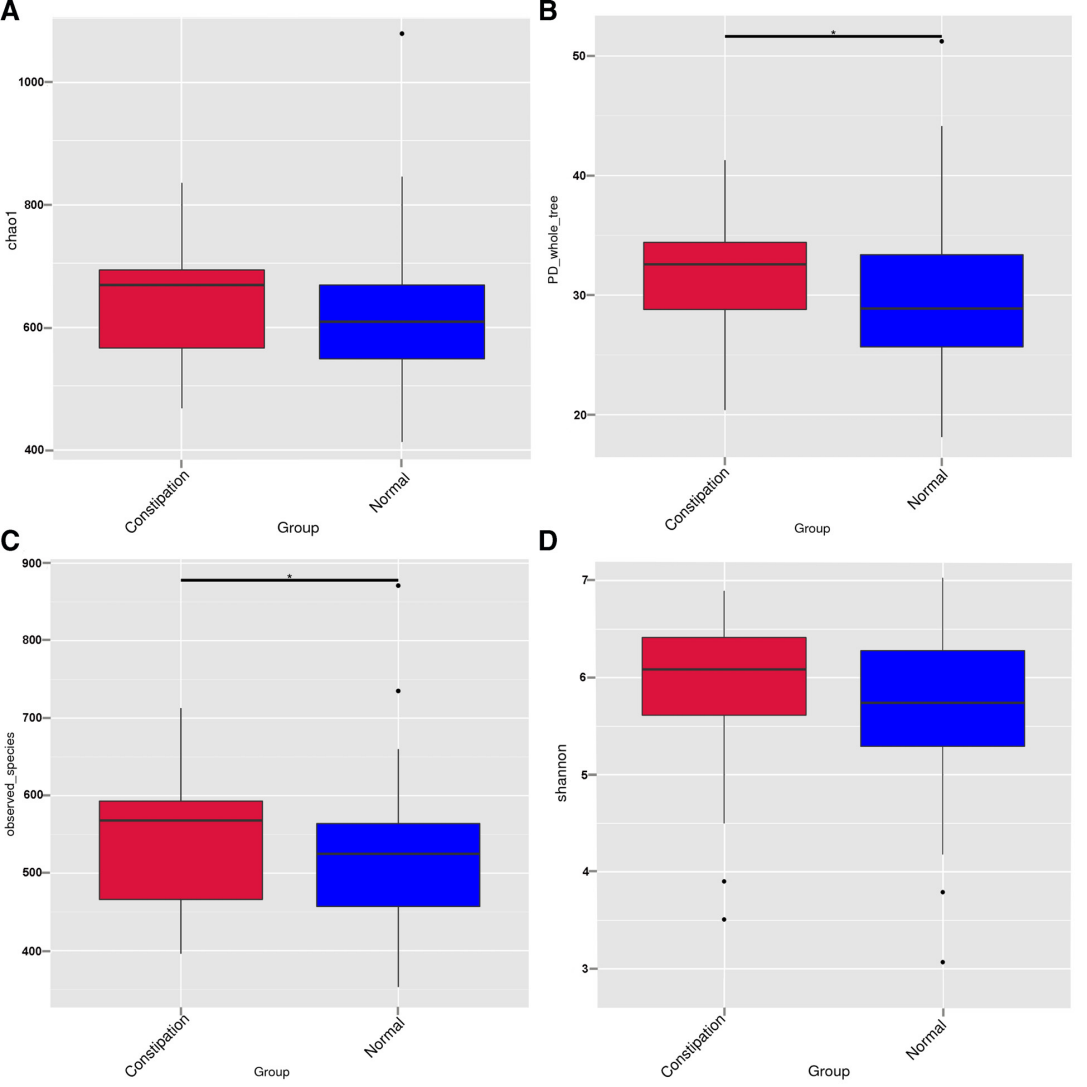
The alpha diversity indices of the fecal microbiota in patients with constipation and normal. (A–D) Box plots depict differences in the fecal alpha diversity indices according to the Chao 1 index, PD whole tree index, observed species index, Shannon index based on the OUT counts. Each box plot represents the median, interquartile range, minimum, and maximum values. *P < .05.

The mean community diversity of the PD whole tree and observed species indices were significantly higher in the constipation group compared with the normal group (all *P *< .05; Figure 6B and C). The Chao1 and Shannon indices were numerically higher in the constipation group compared with the normal group, but the differences were not significant (*P *> .05, Figure 6A and D).

### 3.8. Comparison of beta diversity of the gut microbiota between the constipation and normal groups

**Figure 7.**
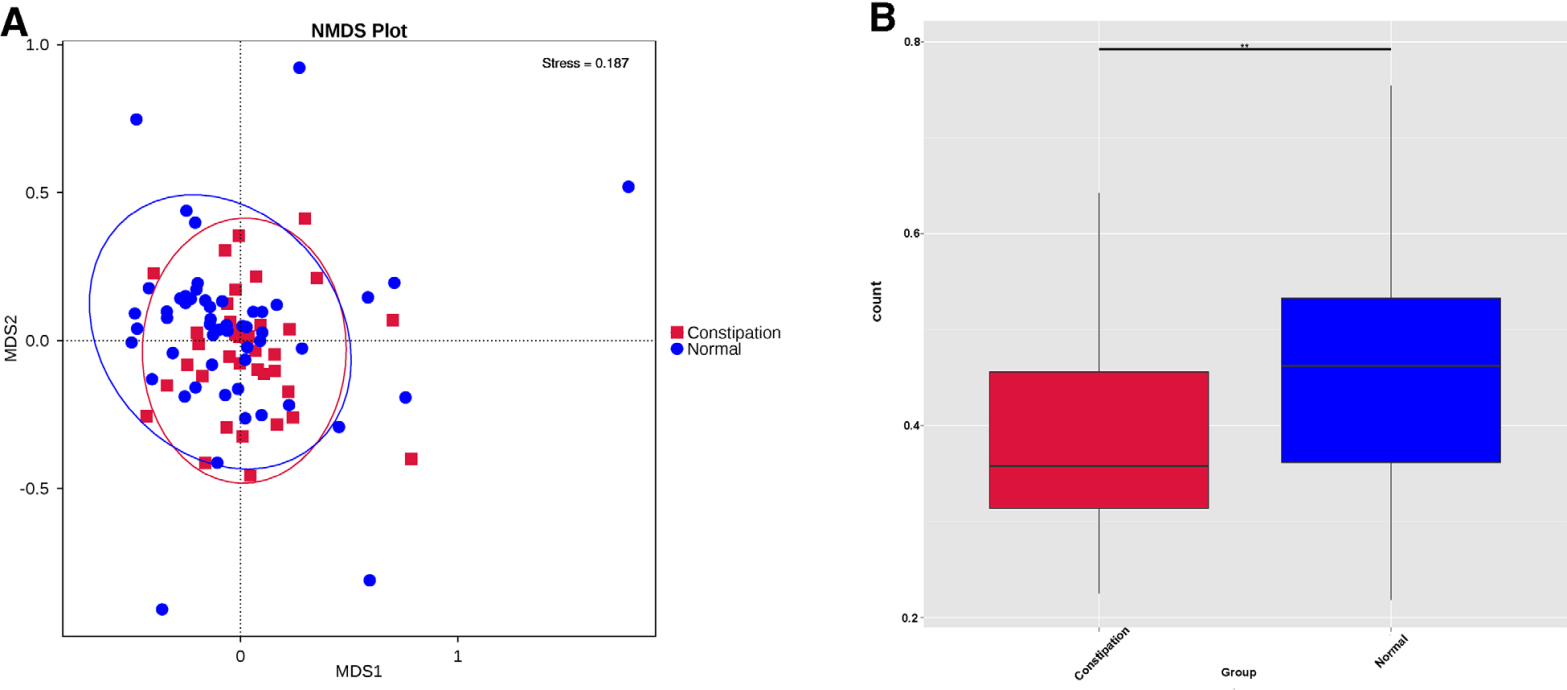
The beta diversity indices of the fecal microbiota in patients with constipation and normal. (A) Non-metric multi-dimensional scaling (NMDS) plot of weighted UniFrac analysis of relative sample OTU composition. NMDS analysis was limited to 2 dimensions, with a stress measurement of 0.187. The stress measurement <0.2 indicated that NMDS could accurately reflect the degree of differences between samples. Each dot represented a scaled measure of the composition of a given participant, and samples from the same group were represented using the same color and shape. (B) Weighted Anosim based on the distance matrix of UniFrac dissimilarity of the fecal microbiota communities in the constipation and normal groups. Each box plot represents the median, interquartile range, minimum, and maximum values. **P < .01.

Beta diversity differed significantly between the constipation and normal groups (Fig. 7A). Further, ANOSIM revealed a significant difference between the constipation and normal groups (ANOSIM *R* = 0.126, *P* < .01, Figure 7B).

### 3.9. Comparison of LEfSe of the gut microbiota between the constipation and normal groups

**Figure 8.**
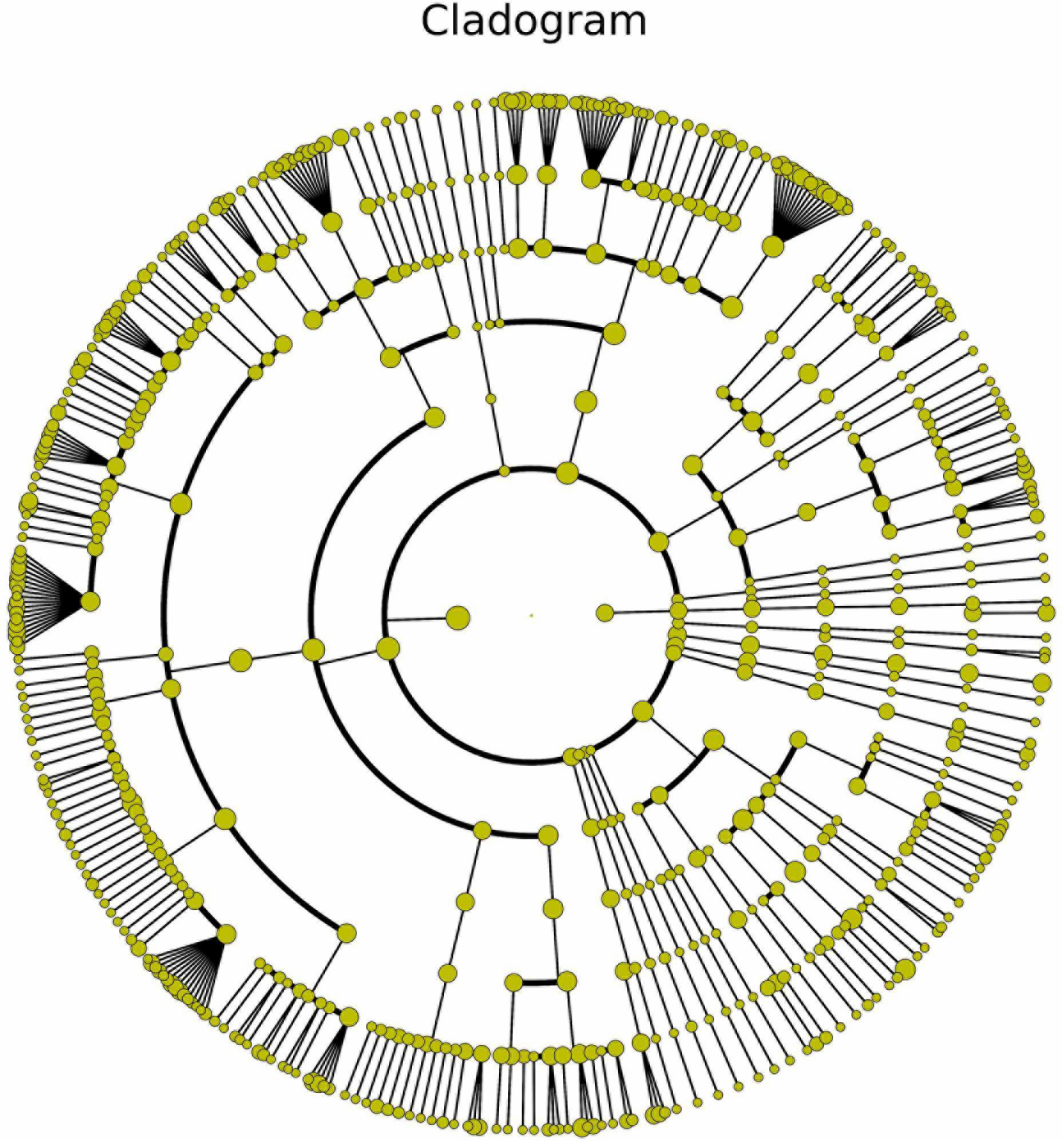
The cladogram illustrates the phylogenetic distribution of microbial lineages between constipation and normal groups. Differently abundant microbiota is listed and marked by different color. The diameter of each circle’s diameter is proportional to the taxon’s abundance.

Based on LEfSe, there was no significant difference in the gut microbiota between the constipation and normal groups (Fig. 8).

### 3.10. Comparison of the gut microbiota genera between the constipation and normal groups

**Figure 9.**
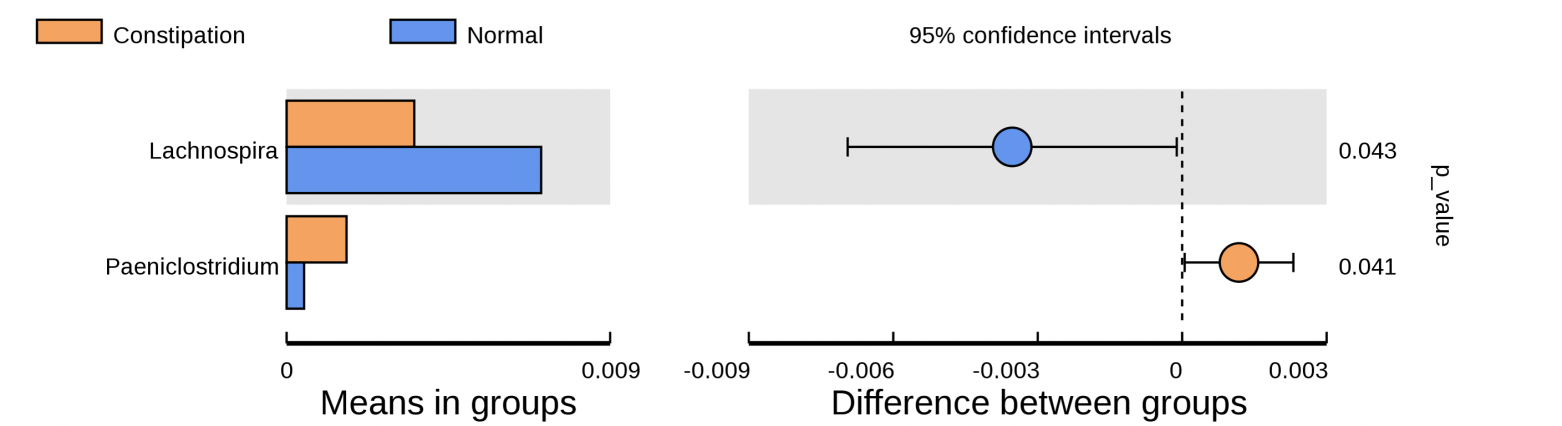
Different fecal microbiota in patients with constipation and normal in genera level. The left histograms represented the mean relative abundance of microbiota in each group, and the right side represented *P* values and the 95% confidence intervals of difference between groups. Significant statistical difference by *t* test (*P* < .05).

We found that *Paeniclostridium* was significantly more abundant in the constipation group (Fig. 9), similarly to the MCI group (Fig. 5C). On the contrary, *Lachnospira* was less abundant in the constipation group compared with the normal group (Fig. 9), similarly to the AD group (Fig. 5A).

## 4. Discussion

The balance of the gut microbiota plays an important role in human health. Increasing evidence has demonstrated that the gut microbiota can influence brain function and behavior via the microbiota–gut–brain axis.^[32]^ Moreover, there seems to be a link between constipation and alterations of the gut microbiota as a risk factor of neurodegenerative disease.^[18]^ In the present study, we investigated the correlation between alterations of the gut microbiota and constipation in older adults with AD or MCI.

Our findings clearly revealed that alpha and beta diversity of the gut microbiota differed significantly between the AD, MCI, and control groups. The AD group showed higher Chao1, PD whole tree, and observed species indices compared with the control group. Similarly, alpha diversity of the microbial community was increased in Japanese patients with AD.^[33]^ However, a previous study found that only the PD whole tree index in the AD group was significantly lower than that of the control group, while the Chao1, PD whole tree, and Shannon indices were not significant differences between the 3 groups.^[11]^ Conversely, several studies have indicated that patients with AD show lower Chao1 and Shannon indices compared with the controls, but no difference in the Simpson index.^[34–36]^ Additionally, some researchers have reported no significant differences in alpha diversity between normal control, MCI, and AD groups.^[4,24,37,38]^ In the present study, the Chao1, Shannon, PD whole tree, and observed species indices did not differ significantly between the AD and MCI groups. We found significantly higher PD whole tree, observed species, and Shannon indices in the MCI group compared with the control group. However, researchers have previously reported that patients with MCI showed a lower Chao1 index compared with the control group, with no significant differences for the Shannon and Simpson indices.^[39,40]^ Regarding beta diversity of the gut microbiota, there were significant differences between the AD, MCI, and control groups. Many previous studies have revealed consistent results,^[11,41]^ but other studies demonstrated no significant overall difference in the beta diversity between the 3 groups.^[24,37]^ The inconsistent results may be due to the sample size, population, dietary habits, lifestyles, RNA sequencing area, comorbidity burden, and the environment in which the participants live. Therefore, it is important to conduct a larger nationwide, multicenter study to obtain more accurate results.

We also divided our 90 participants into a constipation group and a non-constipation (normal) group, and compared the differences in the gut microbiota between the 2 groups. The PD whole tree and observed species indices were significantly higher for the constipation group, and the beta diversity of this group was also significantly different compared with the normal group. These differences indicate that patients with constipation have a notably different gut microbiota composition compared with healthy subjects. Previous studies have also found that the alpha diversity of the gut microbiota in patients with constipation is richer than healthy subjects.^[42]^ Tian et al.^[43]^ reported significantly higher Shannon and Simpson indices of the gut microbiota in the constipation group compared with the control group, and the 2 groups also differed significantly in the gut microbiota composition at different taxonomic levels. It may be inferred that constipation is closely related to alterations of the gut microbiota, and the gut microbiota influences cognitive and neuronal function via the gut–brain axis.

LEfSe revealed significant enrichment of microbial phyla, classes, orders, families, genera, and species between the different groups. At the genus level, *Methanobrevibacter*, *Desulfovibrio*, *Intestinibacter* were significantly more abundant in the AD and MCI groups compared with the control group. Recent studies have found that *Methanobrevibacter* may be associated with an elevated likelihood of developing AD,^[44]^ and most pathways related to cognitive impairment are connected with *Methanobrevibacter*.^[45]^ Excessive proliferation of *Methanobrevibacter* might exacerbate cognitive impairment by inducing oxidative stress through methane. *Desulfovibrio* can produce hydrogen sulfide and lipopolysaccharides, which might induce oligomerization and aggregation of alpha synuclein, thereby affecting the host’s neurocognitive function.^[46]^ *Desulfovibrio* is one of the risk factors for AD, and elevated abundance of this genus increases AD probability.^[47]^ The genus *Intestinibacter* contains only 1 species, *Intestinibacter bartlettii*, which has been reported to be a potentially harmful bacterium in neurodevelopmental disorders and moyamoya disease.^[48]^ A study confirmed that quantification of beta-amyloid deposition at the global and regional brain levels correlates positively and significantly with *Intestinibacter*.^[49]^

Although *Epulopiscium* was significantly elevated in the AD group, there is currently a lack of direct evidence for its relationship with AD. However, its unique metabolic functions, such as polysaccharide fermentation and generation of short-chain fatty acids (SCFAs), suggest that it may indirectly affect neurodegenerative diseases through the gut–brain axis. *Oscillibacter* has been associated with a decreased risk of AD.^[50]^ It may play a protective role in AD through butyric acid synthesis, tryptophan metabolism, and immune regulation, and its reduced abundance is closely related to cognitive decline. However, the specific mechanism requires elucidation. We observed an opposite result (*P* = .03), which may be due to the sample size, population, dietary habits, lifestyles, RNA sequencing area, comorbidity burden, and the environment in which the participants live.

We also found that, compared with the control group, the beneficial genera *Roseburia*, *Lachnospira*, *Fusicatenibacter*, *Lachnoclostridium*, and *Dorea* were significantly less abundant in the AD group, consistent with previous studies.^[51–53]^ *Roseburia*, *Lachnospira*, *Fusicatenibacter*, and *Lachnoclostridium* play a protective role in AD by producing butyric acid.^[54]^ *Dorea* appears to have either pro- or anti-inflammatory functions depending on the surrounding intestinal bacteria and/or the available nutrients. Moreover, *Anaerostipes* was significantly more abundant in the AD and MCI groups compared with the control group. A recent study showed a significant decreasing trend of some genera in the order of the control, MCI, and AD groups, including *Anaerostipes* and *Roseburia*, which are known to produce butyrate and other SCFAs.^[55]^ This indicates that the potential nexus between the gut microbiota and AD may involve modulation of intestinal homeostasis. The present findings suggest that a more pronounced neuroinflammatory status in areas related to cognitive impairment is associated with gut dysbiosis, namely decreased butyrate-producing bacteria.^[55]^

*Paraprevotella* is associated with reduced AD risk.^[47]^ In the present study, *Paraprevotella* in the AD group and *Prevotellaceae* in the MCI group were significantly less abundant compared with the control group. *Prevotellaceae* is the predominant bacterial family in the human gut; it includes multiple genera, such as *Prevotella* and *Paraprevotella*. Higher abundance of *Paraprevotella*, the members of which degrade trypsin in the gut, reduces inflammation; protects the gut; and increases acetic, propionic, butyric, isobutyric, and valeric acids as well as total SCFAs in fermented feces.^[56]^ A decrease in *Prevotellaceae* and *Prevotella* may increase intestinal inflammation via dysregulated mucin biosynthesis, which can influence various signals related to AD pathogenesis.^[57]^

The *Clostridium* genus belongs to the phylum *Firmicutes* and contains species that are the causative agents of pathogenic processes in humans. *Clostridiaceae* is an important family under the order *Clostridiales* and includes the genera *Clostridium* and *Paeniclostridium*. In the present study, *unidentified Clostridiales* was significantly more abundant in the MCI group compared with the control group. On the contrary, the authors of a previous study reported a higher abundance of *Clostridium sensu stricto 1* in participants with better cognitive function,^[37]^ while in another study, the authors noted a decrease in *Clostridium butyricum* in the participants with obesity, positing that it may contribute to cognition decline.^[58]^ In addition to SCFAs, members of *Clostridiaceae* produce indole propionic acid, an antioxidant that protects primary neurons and neuroblastoma cells against beta-amyloid.^[34,37]^ The inconsistent research results may be due to differences in the sample size, population, and RNA sequencing area and methods.

Although *Cellulosilyticum* and *Peptococcus* were significantly more abundant in the MCI group, there is relevant rare association between them and neurological and psychiatric disorders. Therefore, further study is needed to determine the relationship between *Cellulosilyticum* and *Peptococcus* and MCI. The authors of a Spanish study showed that in the middle stages of cognitive impairment in AD, coinciding with dementia development (i.e., stage 4 of the Global Deterioration Scale), *Peptococcus* is related to heptanoic acid.^[59]^ Members of *Butyricimonas*, which are protective, can produce butyric acid and isoacid salts.^[60]^ According to previous studies, *Butyricimonas* is inversely associated with cognitive impairment,^[60,61]^ but we found that it was significantly more abundant in the MCI group. Additionally, the relative abundance of *Actinomyces* was increased in the MCI group compared with the control group. The authors of a previous study reported that *Actinomyces* correlates positively and significantly with the Montreal Cognitive Assessment and MMSE scores.^[62]^ Our results may be inconsistent due to differences in sample size, population, and RNA sequencing regions and methods.

Remarkably, we found that *Lachnospira* was significantly less abundant in the constipation group compared with the normal group; the AD group also showed a lower abundance of this genus compared with the control group. Constipation might affect brain function and behavior by altering the gut microbiota, ultimately leading to a range of CNS diseases.^[15,17]^ In a previous study, researchers reported a greater than 4-fold higher level of *Lachnospira* in healthy subjects compared with constipated subjects.^[63]^ Several members of *Lachnospira* are capable of producing lactate and acetate, and lactate may be further metabolized into butyrate or propionate. Butyrate can cross the blood–brain barrier to influence activity in the brain and affect microglial function and development. Sodium butyrate decreases neuroinflammation and enhances neuroprotective function on cortical neurons.^[54]^ Decreased abundance of *Lachnospira* reduces the concentration of SCFAs, leading to an aggravated inflammatory response in the body as well as impaired microglial function, increasing the risk of AD.^[41,63]^

We also found a higher abundance of *Paeniclostridium* in the constipation group, similarly to the MCI group. However, the relationship between *Paenicostridium* and MCI is not yet clear. *Paeniclostridium sordellii*, previously known as *Clostridium sordellii*, is a gram-positive pathogen. Based on its potential pathogenicity, *Paenicostridium* might exacerbate neurodegenerative diseases through toxin release. In the future, further study is needed to validate changes in *Paenicostridium* abundance in patients with AD or MCI.

In this study, some limitations should be acknowledged. First, the generalizability of this study was limited due to the single-center, cross-sectional design, small sample size of the study. Multicenter research with a longitudinal design and larger sample size is needed to validate the results. Second, AD and MCI in this study were diagnosed mainly based on the clinical criteria rather than pathological evidence. Although the comprehensive neuropsychological tests of each participant were performed by the expert panel consisting of 2 neurologists and neuropsychologists, future research incorporating PET scanning or Cerebrospinal fluid analysis is still warranted to assess the association between gut microbiota and pathological change. Third, while 16S rRNA sequencing provides robust taxonomic data, its resolution is lower than shotgun metagenomic sequencing.

## 5. Conclusions

In summary, the findings in this study demonstrated that the AD participants had the highest proportion of constipation, and followed by MCI participants and the lowest in the control group. There were significant differences in alpha and beta diversity of the gut microbiota among the AD, MCI and control groups, as well as between the constipation and normal groups. At the genus level, the relative abundance of *Lachnospira* was decreased in both the AD group and the constipation group, while the relative abundance of *Paeniclostridium* was increased in both the MCI group and the constipation group. Our findings suggest that changes in the gut microbiota in patients with AD or MCI are partially consistent with those observed in older adults with constipation and may be involved in the pathogenesis of AD. Future research should extend these findings through longitudinal design and larger sample size studies to validate the results.

## Acknowledgments

The authors are grateful to the participants and staff of this study. The authors would like to thank the Natural Science Foundation of Guangxi, the Key Laboratory of Human Development and Disease Research (Guangxi Medical University), Education Department of Guangxi Zhuang Autonomous Region, the Fudan Professorship Initiating Grant, the Natural Science Foundation of China, and the College Students’ Innovative Entrepreneurial Training Plan Program for funding this study.

## Author contributions

**Data curation:** Kai-Yong Huang, Liu-Chun He, Lan-Shu Zhou.

**Formal analysis:** Kai-Yong Huang, Lan-Shu Zhou.

**Funding acquisition:** Kai-Yong Huang, Shu-Ting Zhang, Jing Zhou, Lan-Shu Zhou, Guo-Dong Lu.

**Investigation:** Kai-Yong Huang, Yi-Yu Wei, Shu-Ting Zhang, Liu-Chun He.

**Methodology:** Kai-Yong Huang, Yi-Yu Wei, Shu-Ting Zhang, Guo-Dong Lu.

**Validation:** Yi-Yu Wei, Jing Zhou.

**Software:** Liu-Chun He, Jing Zhou.

**Supervision:** Liu-Chun He.

**Conceptualization:** Hua-Bin Su, Guo-Dong Lu.

**Project administration:** Hua-Bin Su, Guo-Dong Lu.

**Writing – original draft:** Kai-Yong Huang, Yi-Yu Wei, Shu-Ting Zhang.

**Writing – review & editing:** Hua-Bin Su, Guo-Dong Lu.
